# Robotic multiquadrant colorectal procedures: A single-center experience and a systematic review of the literature

**DOI:** 10.3389/fsurg.2022.991704

**Published:** 2022-08-17

**Authors:** Giorgio Bianchi, Paschalis Gavriilidis, Aleix Martínez-Pérez, Gian Luigi de’Angelis, Mathieu Uzzan, Iradj Sobhani, Federico Coccolini, Carlo Alberto Schena, Maria Clotilde Carra, Giuseppe Spinoglio, Nicola de’Angelis

**Affiliations:** ^1^ Unit of general surgery, CARE Department, Henri Mondor University Hospital, Créteil, France; ^2^ Department of medicine and surgery, University of Parma, Parma, Italy; ^3^ Gastroenterology and Endoscopy Unit, Department of Medicine and Surgery, University Hospital of Parma, Parma, Italy; ^4^ Department of surgery, University Hospitals Coventry and Warwickshire NHS Trust, Coventry, United Kingdom; ^5^ Faculty of Health Sciences, Valencian International University, Valencia, Spain; ^6^ Department of General and Digestive Surgery, Hospital Universitario Doctor Peset, Valencia, Spain; ^7^ Department of Gastroenterology, APHP-Henri Mondor University Hospital, Creteil, France; ^8^ EC2M-EA7375 Research Team, Henri Modor Campus, Paris East University, Creteil, France; ^9^ General, Emergency and Trauma Department, Pisa University Hospital, Pisa, Italy; ^10^ Rothschild Hospital, AP-HP, Université de Paris, Paris, France.; ^11^ IRCAD Faculty Member Robotic and Colorectal Surgery-IRCAD, Strasbourg, France; ^12^ University Paris-Est, UPEC, Créteil, France

**Keywords:** total proctocolectomy, ileal pouch-anal anastomosis, total colectomy, robotic surgery, ulcerative colitis, familial adenomatous polyposis

## Abstract

**Purpose:**

Robotic surgery has been progressively implemented for colorectal procedures but is still limited for multiquadrant abdominal resections. The present study aims to describe our experience in robotic multiquadrant colorectal surgeries and provide a systematic review and meta-analysis of the literature investigating the outcomes of robotic total proctocolectomy (TPC), total colectomy (TC), subtotal colectomy (STC), or completion proctectomy (CP) compared to laparoscopy.

**Methods:**

At our institution 16 consecutive patients underwent a 2- or 3-stage totally robotic total proctocolectomy (TPC) with ileal pouch-anal anastomosis. A systematic review of the literature was performed to select studies on robotic and laparoscopic multiquadrant colorectal procedures. Meta-analyses were used to compare the two approaches.

**Results:**

In our case series, 14/16 patients underwent a 2-stage robotic TPC for ulcerative colitis with a mean operative time of 271.42 (SD:37.95) minutes. No conversion occurred. Two patients developed postoperative complications. The mean hospital stay was 8.28 (SD:1.47) days with no readmissions. Mortality was nil. All patients underwent loop-ileostomy closure, and functional outcomes were satisfactory. The literature appraisal was based on 23 retrospective studies, including 736 robotic and 9,904 laparoscopic multiquadrant surgeries. In the robotic group, 36 patients underwent STC, 371 TC, 166 TPC, and 163 CP. Pooled data analysis showed that robotic TC and STC had a lower conversion rate (OR = 0.17;95% CI, 0.04–0.82; *p* = 0.03) than laparoscopic TC and STC. The robotic approach was associated with longer operative time for TC and STC (MD = 104.64;95% CI, 18.42–190.87; *p* = 0.02) and TPC and CP (MD = 38.8;95% CI, 18.7–59.06; *p* = 0.0002), with no differences for postoperative complications and hospital stay. Reports on urological outcomes, sexual dysfunction, and quality of life were missing.

**Conclusions:**

Our experience and the literature suggest that robotic multiquadrant colorectal surgery is safe and effective, with low morbidity and mortality rates. Nevertheless, the overall level of evidence is low, and functional outcomes of robotic approach remain largely unknown.

**Systematic Review Registration:**

https://www.crd.york.ac.uk/prospero/, identifier: CRD42022303016.

## 1. Introduction

In the last two decades, robotic surgery has been progressively implemented as a minimally invasive approach for colorectal procedures (1, 2). Nowadays, robotic colorectal surgery is widely used, and evidence supports its safety and efficacy compared to laparoscopic surgery (3, 4). Despite its large applications (5, 6), there is limited data on the use of robotic surgical platforms for extended colorectal resections requiring access in all four quadrants of the abdomen (7) mainly because, to achieve this by robotics, a repositioning of the patient-side robotic cart is needed, leading to an increased operative time and workload (7, 8). The last generation of robotic platforms allows the rotation of the robotic cart without the need for robot repositioning, and thus it may favor the performance of multiquadrant procedures (7, 9).

Four colorectal procedures require a multiquadrant approach, including subtotal colectomy (STC) (10), total colectomy (TC) (11), total proctocolectomy (TPC) (12), and completion proctectomy (CP) (11), performed in a staged multiquadrant surgery. TPC with ileal pouch-anal anastomosis (IPAA) (11, 13), which avoids permanent stoma (12), can be performed in one- (11), two- (11, 14), or three- (11, 14) stage surgery, depending on the extent of the resection and the performance of a loop ileostomy, which will finally require a reversal procedure.

The present study aims to describe our experience in robotic multiquadrant colorectal surgeries (particularly TPC and CP) using the da *Vinci Xi* (Intuitive Surgical Inc, Sunnyvale CA, USA) robotic platform. In addition, we conducted a systematic review and meta-analysis of the literature to investigate the outcomes of robotic multiquadrant colorectal surgeries and compare them with those associated with laparoscopy.

## 2. Material and methods

### 2.1. Single-center experience

#### 2.1.1. Study population

We identified consecutive adult patients who underwent a 2- or 3-stage robotic TPC with IPAA between January 2014 and December 2021 in elective settings in a prospectively maintained database at a tertiary care center (Henri Mondor University Hospital of Creteil, France).

A robotic TPC was defined as all procedural stages performed robotically using *da Vinci Xi* (Intuitive Surgical Inc, Sunnyvale CA, USA).

Indication for surgery was established in the institutional multidisciplinary meeting discussion for all patients.

The patient is placed in a modified lithotomy position with the legs in Allen stirrups. The robotic cart is placed between the patient's legs on the median axis. This positioning allows the rotation of the boom in all necessary positions as well as an easy access for the surgeon for the introduction of circular stapler. Only the robotic boom is rotated and manually adjusted in two different positions according to the operational steps: (1) right colectomy and transverse colon mobilization; (2) left colectomy and rectal resection with total mesorectal excision (TME) and IPAA. In total, 4 robotic (three 8-mm ports and one 12-mm port) and 2 laparoscopic (5-mm) ports are used. The 4 robotic ports are placed on a diagonal line drawn from the right femoral head (lateral border of the inguinal triangle) to where the left mid clavicular line (MCL) crosses over the left subcostal margin. The two laparoscopic ports are placed 2 cm laterally of the MCL at the level of the left and right subcostal margin.

In the two-stage procedure, the TPC is performed following a medial-to-lateral approach. The procedure starts from the right side; a complete mobilization of right and transverse colon, and the splenic flexure is performed with the proximal ligation of the ileo-colic, right and middle colic pedicles. After rotation of the robotic boom, the left colectomy is performed with a medial-to-lateral approach with the ligation of the inferior mesenteric artery and vein. Last step of the dissection consists of a sphincter-preserving low rectum resection with TME. The rectal transection is achieved with Endo-Wrist 45-mm stapler. The surgical specimen is extracted by a 3 cm incision right to the umbilicus. A 20-cm ileal J pouch is done extracorporeally using GIA 80-mm stapler, the anvil of circular 29-mm stapler is fixed with purse-string suture in the distal angle of the J pouch and an ileo-anal end-to-end anastomosis is constructed with a circular 29-mm stapler introduced transanally. A protective loop ileostomy is exteriorized using the incision close to the umbilicus.

In the first part of the three-stage procedure, the total colectomy is performed with the same technique. Once the colon is totally mobilized, following exsufflation, an incision is made 3 cm left to the umbilicus; this incision is used for extracting the colon, the ileum is dissected using GIA 80-mm stapler 5 cm proximally from the ileo-cecal valve; the sigmoid colon is dissected proximal to the sacral promontory. A double-barreled ileo-sigmoid ostomy (15–17) is created.

In the second operation, the ileo-sigmoid ostomy is dissected, the robotic cart is placed on the left side of the patient and the rectal resection with TME and IPAA are performed using the same technique of the two-stage technique. A protective loop ileostomy is exteriorized using the incision close to the umbilicus.

A more detailed description of the surgical procedures and operative techniques is provided as **Supplementary Material**.

Data collected included patient demographics (age, sex), previous abdominal surgery, indication for operation, American Society of Anesthesiologists (ASA) score, operative clinical variables (operative time, blood loss, conversion to laparoscopy or open surgery), postoperative outcomes (postoperative complications, 90-days morbidity and mortality, hospital stay, rate of stoma closure), and assessment of fecal incontinence, using the Wexner score (18). The Dindo-Clavien classification was used to define and grade postoperative complications (19).

Data are presented as frequencies and percentages for categorical variables and mean and standard deviation for continuous variables. This retrospective study exclusively used clinical record data routinely collected in health databases (MR004 regulation) and declared to the National Commission for Data Protection and Liberties (CNIL: 2210699). All personal data were collected after obtaining informed consent, and patients received treatment following the ethical standards of the Helsinki Declaration.

### 2.2. Systematic review

A systematic review of the literature was performed following the Cochrane Collaboration specific protocol (20) and reported according to the Preferred Reporting Items for Systematic Reviews and Meta-Analyses (PRISMA) statement (21) (Additional Tables 1, 2). The protocol was registered in PROSPERO, University of York (Id: CRD42022303016)

**Table 1:** Patients’ characteristics and surgical outcomes.

Patients’ characteristics	Robotic procedures (*n* = 16)
Age (years) [mean (SD)]	44.42 (3.73)
Female [*n* (%)]	3 (18.7)
ASA I–II [*n*]	10/6
Previous abdominal surgery [*n* (%)]	3 (18.7)
Diagnosis [*n* (%)]	
Ulcerative colitis (UC)	14 (87.5)
Familial adenomatous polyposis (FAP)	2 (12.5)
Surgical outcomes	
Robotic Surgical procedure [*n* (%)]	
Total Proctocolectomy with IPAA / loop ileostomy	14 (87.5)
Total Colectomy with ileosigmoidostomy / Proctocolectomy with IPAA / loop ileostomy	2 (12.5)
Operative time (min) [mean (SD)]	271.42 (37.95)
Blood loss [mean (SD)]	128.57 (43.23)
Conversion to open or laparoscopic surgery [*n*(%)]	0
Dindo-Clavien complication ≥III [*n*(%)]	2 (12.5)
Anastomotic leak [*n*(%)]	2 (12.5)
Reoperation [*n* (%)]	0
Morbidity at 90 days [*n* (%)]	3 (18.7)
Mortality at 90 days [*n* (%)]	0
Hospitality stay (days) [mean (SD)]	8.28 (1.47)
Re-Hospitalization [*n* (%)]	0
Loop ileostomy closure [*n* (%)]	16 (100)
Wexner score^a^ [*n* (%)]	
Asymptomatic	9 (56.5)
Moderate symptoms	5 (31)
Severe symptoms	2 (12.5)

IPAA, ileal pouch-anal anastomosis.

^a^ Assessed at 3 months after stoma closure.

**Table 2:** Overview of the included studies.

Reference (First author, Year)	Study design, Time frame	Patients undergoing multiquadrant surgery / Total patients	Type of interventions	Main outcomes	Principle findings
Comparative studies—Robotic surgery vs. Laparoscopic surgery					
Anvari et al. 2004 (28)	Retrospective cohort 2002–2003	2/20	All types colectomies	Perioperative outcomes	Robotic colectomy for benign and malignant disease is safe and feasible.
D’Annibale et al. 2004 (2)	Retrospective cohort 2001–2003	3/106	Colorectal procedures	Perioperative outcomes	Robotic and laparoscopic techniques can achieve the same operative and postoperative results
Spinoglio et al. 2008 (29)	Retrospective cohort 2005–2007	4/211	Colorectal resection	Perioperative outcomes	Robotic colon surgery is feasible and safe. A longer operating time is needed.
Byrn et al. 2012 (30)	Retrospective cohort (A) NR	2/14	Single incision colectomy	Perioperative outcomes	Single-incision robotic colectomy is safe and feasible
Miller et al. 2012 (31)	Retrospective matched case-control 2009–2010	34/34	Proctectomy	Short-term and functional outcomes	Robotic proctectomy is a safe and effective technique for patients with IBD. It is comparable to laparoscopic proctectomy with regard to perioperative outcomes, complications, and short-term functional results.
Helvind et al. 2013 (32)	Retrospective case-control 2009–2012	3/263	Colonic resection	Perioperative outcomes	Robot colonic resection is a safe and feasible alternative to traditional laparoscopic resection for colonic cancer.
Mark-Christensen et al. 2016 (33)	Retrospective cohort 2004–2014	81/251	Restorative proctectomy and proctocolectomy^a^	Early postoperative outcome	Robot IPAA appears to be a safe alternative to open IPAA surgery, offering comparable short-term outcome although associated with a longer duration of operation and higher readmission rates.
Moghadamyeghaneh et al. 2016 (34)	Retrospective cohort on a national database (NIS) 2009–2012	9,940/26,721	Total colectomy^b^	Perioperative outcomes	Minimally invasive approaches to total colectomy are safe, with the advantage of lower mortality and morbidity compared to an open approach. Robotic surgery had a significantly lower conversion rate compared to laparoscopic approach. Total hospital charges are significantly higher in robotic surgery compared to laparoscopic approach.
Rencuzogullari et al. 2016 (35)	Retrospective cohort 2010–2014	42/42	Restorative proctectomy and proctocolectomy	Perioperative outcomes, long-term functional outcome and QOL	Despite longer operative time and higher estimated blood loss compared with laparoscopic proctectomy, robotic proctectomy provided similar short- term postoperative results, long-term functional outcomes, and QOL of life in this study.
Jimenez-Rodriguez et al. 2018 (7)	Retrospective cohort 2015–2017	23/23	Total colectomy	Perioperative outcomes	The da Vinci Xi robotic platform may overcome some of the disadvantages of older-generation platforms and is associated with similar operative time for this specific complex colorectal operation.
Marino et al. 2018 (36)	Retrospective matched case-control (A) 2014–2017	32/32	Restorative proctocolectomy	Short-term outcomes	Although the costs for robotic proctectomy are high, the technique allows reducing the estimated blood loss and conversion rate overcoming some limitations of laparoscopic surgery both in ergonomic and accuracy aspects.
Elias et al. 2019 (37)	Retrospective matched case-control (A) 2008–2017	105/105	Restorative proctectomy and proctocolectomy	Perioperative outcomes	Robotic surgery enables superior total mesorectal excision and distal transection with elimination of the at-risk rectal cuff with improved postoperative outcomes in patients undergoing IPAA for ulcerative colitis and familial adenomatous polyposis.
Lightner et al. 2019 (38)	Retrospective cohort 2015–2018	132/132	Restorative proctectomy and proctocolectomy	30-day postoperative outcome	Laparoscopic and robotic IPAA have equivalent postoperative morbidity
Ozben et al. 2019 (39)	Retrospective cohort 2010–2018	82/82	Total/subtotal abdominal colectomy	30-day perioperative outcomes	In total/subtotal colectomy procedures, the robotic approach with the da Vinci Xi platform is feasible, safe, and associated with short-term outcomes similar to laparoscopy but longer operative times and a higher number of retrieved lymph nodes.
Kim et al. 2021 (40)	Retrospective cohort 2017–2021	56/56	Total colectomy and proctocolectomy	Operative and post-operative outcomes	The advantages of the boom system and motion- sensitive table were successfully utilized to integrate anatomical dissection with the multiquadrant procedures of TC/TPC, with none of these patients requiring conversion to open surgery.
Case series—Robotic surgery					
Zimmern et al. 2010 (1)	Retrospective case series 2005–2009	7/131	Colorectal resections	Perioperative outcomes	Robotic colon and rectal resections are safe and feasible options for the treatment of both benign and malignant disease processes.
Pedraza et al. 2011 (41)	Prospective case series 2008–2010	6/5	Restorative proctocolectomy	Perioperative outcomes	Robotic surgery is a safe and feasible approach for those with ulcerative colitis requiring surgery with restoration of bowel continuity.
Domajnko et al. 2012 (42)	Retrospective case series (A) 2008–2010	27/27	Restorative proctectomy and proctocolectomy	Perioperative and functional outcomes	Robotic-assisted IPAA is a safe and feasible technique. Although the learning curve is steep, robotic technology is a valuable tool for pelvic surgery.
McLemore et al. 2012 (43)	Retrospective case series 2010–2012	3/3	Restorative proctectomy	Perioperative and functional outcomes	In this series of patients with toxic ulcerative colitis, the robotic platform was found to be a feasible and safe approach for pelvic dissection during the stage II procedure: completion proctectomy with ileoanal pouch reconstruction.
Morelli et al. 2015 (44)	Retrospective case series 2010–2014	6/6	Hand-assisted hybrid laparoscopic–robotic restorative total proctocolectomy	Perioperative and functional outcomes	Hybrid hand-assisted laparoscopic–robotic proctocolectomy with IPAA is an appealing alternative to laparoscopy and open surgery in selected patients with FAP or UC.
Roviello et al. 2015 (45)	Case series 2014	4/4	Proctocolectomy	Perioperative outcomes	Robotic single docking technique for total proctocolectomy for UC shows that this procedure is safe and feasible when performed by robotic experienced surgical team.
Hamzaoglu et al. 2019 (46)	Retrospective case series 2015–2017	10/10	Restorative proctocolectomy	Perioperative outcomes	Totally robotic restorative proctocolectomy is a safe and feasible option for the surgical treatment of UC. The Xi robotic platform facilitates multiquadrant surgery and enables to remove colon and rectum with a totally robotic technique in the same setting of a RP/IPAA procedure.
Hollandsworth et al. 2020 (47)	Case series 2016–2019	37/37	Subtotal colectomy and proctocolectomy	Perioperative outcomes	The described technique is a safe approach for multiquadrant robotic colorectal surgery given the low rate of associated morbidity and mortality and has a reasonable learning curve for experienced colorectal surgeons.

QOL, Quality of Life; IBD, Inflammatory Bowel Disease; IPAA, ileal-pouch anal anastomosis; FAP, familial adenomatous polyposis; UC, ulcerative colitis; RP, restorative proctectomy; (A), Conference abstract.

^a^ Open surgery as comparison rather than laparoscopic.

^b^ Laparoscopic and open surgery as comparison.

#### 2.2.1. Study selection criteria

We defined the study selection criteria before data collection to identify eligible studies. All publications describing the efficacy, safety, complications, and outcomes of robotic multiquadrant colorectal procedures were retrieved and analyzed. Due to the expected paucity of studies in the literature, comparative and non-comparative studies (case series) were both considered.

The research equations were built according to the following PICOS format:

*P, population*: patients who required multiquadrant colorectal procedures.
*I, intervention*: robotic or robot-assisted TC, STC, TPC, CP performed as one-, two- or three-stage procedures.
*C, comparisons*: laparoscopic multiquadrant colorectal procedures, or no comparison.
*O, outcome(s):* operative, postoperative, and long-term surgical outcomes, including functional outcomes.
*S, study design:* comparative studies (e.g., case-control) and case series. Published articles and conference abstracts were eligible for inclusion.

Single case reports and review articles were excluded. Studies on open surgery procedures may be eligible if separated outcomes of interest were reported for the robotic or the laparoscopic approaches.

No trial duration limitation was set. No publication date restriction was applied but only English literature was considered.

#### 2.2.2. Literature search strategy

A literature search was performed using the following online databases up to January 2022: Medline (through PubMed), Embase, and Cochrane Library. A specific research query was formulated for each database, using the following keywords and MeSH terms: robotic surgery; robotics; robot-assisted; minimally invasive surgery; colectomy; total colectomy; subtotal colectomy; proctocolectomy; completion proctectomy; large bowel resection; total proctocolectomy; restorative proctocolectomy; completion proctocolectomy; ileal pouch-anal anastomosis; IPAA.

We cross-checked reference lists from eligible studies and relevant review articles to identify additional publications.

#### 2.2.3. Article selection and quality assessment

The literature search and selection were performed by two independent reviewers (GB and NdeA). First, all records from the merged searches were reviewed for relevance on title and abstract. Records excluded by both reviewers were removed; any disagreement was resolved by discussion or the intervention of a tiebreaker (GS). Both reviewers performed an independent full-text analysis for the final inclusion or exclusion of pre-selected articles.

The reviewers independently assessed the risk of bias using appropriate tools according to the study design. The Newcastle–Ottawa Scale (NOS) (22) was used for case-control and cohort studies. The case series studies were evaluated using the tool described by Murad et al (23). The Grading of Recommendations Assessment Development and Evaluation (GRADE) system was used to grade the “body of evidence” arising from this review (24).

#### 2.2.4. Data extraction and data analysis

Data from the included studies were processed for qualitative and quantitative analysis. Outcome measures (percentages, mean/median values with standard deviations/ranges) were extracted for each surgical approach. Mean values or standard deviation (SD) values (if not reported) were estimated from the median, ranges, inter-quartile ranges (IQR), or p-values, whenever possible (25, 26). Whenever a meta-analysis was possible to compare robotic multiquadrant procedures vs. laparoscopic procedures, the odds ratio (OR) and 95% CI were estimated using the Mantel–Haenszel method for binary outcome data. The mean differences (MD) and 95% CIs were estimated using inverse variance weighting for continuous data. Heterogeneity was assessed using the *I*^2^ statistic, and values of 25%, 50%, and 75% were considered low, moderate, and high, respectively (25, 27). Random-effects models were used to explore potential inter-study heterogeneity. The effect was considered significant at *p* < 0.05. The meta-analysis was performed using RevMan software (version 5.4; Cochrane Collaboration 2020, Copenhagen, Denmark).

## 3. Results

### 3.1. Single center experience

Between January 2014 and December 2021, 16 consecutive patients underwent robotic TPC with IPAA. The clinical and surgical characteristics of the series are described in Table 1. Patients were relatively young (mean age: 44.42 (SD: 3.37) years) and predominantly male. The most frequent indication for surgery was ulcerative colitis (UC).

Fourteen patients underwent a totally robotic two-stage TPC with IPAA, and 2 underwent a totally robotic three-stage procedure with a TC and a double-barreled ileo-sigmoido ostomy (first step), a CP with IPAA and a loop ileostomy (second step), followed by an ileostomy reversal (third step). The mean operative time was approximately 271 min with no conversions to laparoscopy or open approach. Two patients (12.5%) with a Dindo-Clavien ≥ III complication (i.e., anastomotic leak) were treated with percutaneous drainage and intravenous antibiotics in the postoperative period. The mean hospital stay was >8 days. Mortality was nil. The last surgical stage (loop ileostomy closure) was completed in all patients. The Wexner score for fecal incontinence was assessed at 3 months after ileostomy closure: 9 (56.5%) patients were asymptomatic (0–4), 5 (31%) had moderate symptoms (5–9), and 2 (12.5%) had severe symptoms.

### 3.2. Systematic review and meta-analysis

#### 3.2.1. Literature search and selection

**Figure 1:**
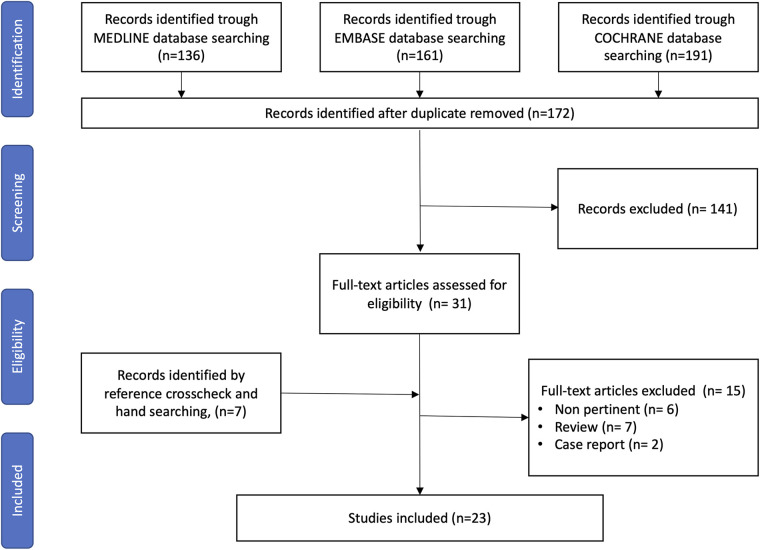
PRISMA flow diagram for study search, selection, inclusion, and exclusion. Example of the research strategy: ((robotic[Title/Abstract])) OR (robotic surgery[Title/Abstract])) OR (robotics[Title/Abstract])) OR (robotic procedure[Title/Abstract])) OR (minimally-invasive surgery[Title/Abstract]))) AND ((((((((((((((((total proctocolectomy[Title/Abstract]) OR (subtotal colectomy[Title/Abstract])) OR (total colectomy[Title/Abstract])) OR (completion proctectomies[Title/Abstract])) OR (completion proctectomy[Title/Abstract])) OR (ileal pouch-anal anastomosis[Title/Abstract])) OR (multiquadrant surgery[Title/Abstract])) OR (multiquadrant abdominal surgery[Title/Abstract])) OR (loop ileostomy[Title/Abstract])) OR (restorative proctocolectomy[Title/Abstract])) OR (restorative coloproctectomy[Title/Abstract])) OR (proctocolectomy[Title/Abstract])) OR (Coloproctectomy[Title/Abstract]))).

After removing duplicates, the literature search yielded 172 articles for screening on title and abstract. Of these, 31 were selected for a full-text evaluation and 7 additional studies were identified by crosschecking reference lists. A total of 23 studies (including 4 conference abstracts) fulfilled the selection criteria and were finally included in this systematic review (Figure 1).

#### 3.2.2. Study characteristics

The 23 selected studies were published between 2004 and 2021; they consisted of 15 cohort studies (2, 7, 28–40) and 8 case series (1, 41–47). All had a retrospective design. One study was based on data from nationwide databases (NIS) (34), and all the others were based on single-center experience. The characteristics of the selected studies are summarized in Table 2.

Of the comparative studies, ten papers and three conference abstracts compared robotic and laparoscopic procedures; three papers were matched case-control studies (31, 36, 37), and ten were cohort studies (2, 7, 28–30, 32–35, 38–40). One cohort study compared robotic to open procedures (33), and another compared robotic with laparoscopic and open procedures (34).

For comparatives and case series studies, we considered short-term postoperative outcomes and observed a 30-day postoperative period of follow-up in all studies. Two cohort studies (31, 35) and two case series (42, 44) reported functional outcomes. Only one cohort study described the quality of life in patients (35).

Overall, the included studies analyzed a total of 28,315 patients undergoing colorectal surgical procedures between 2001 and 2020; of these, 10,640 patients underwent a multiquadrant surgery (736 by robotic and 9,904 by laparoscopy).

In the robotic group, 36 patients underwent an STC, 371 a TC, 166 a TPC, and 163 a CP. The main surgical indication for robotic surgery was UC, concerning more than 50% of the patients; other indications included colorectal cancers and benign tumors in 18.6% and 8%, respectively. The main indications for laparoscopy were UC, colorectal cancers, and functional disorders in 31.8, 18.9 and 14.3% of patients, respectively (Table 3).

**Table 3:** Included study characteristics.

Study Characteristics	Sample size (*n*)				Number and Type of operation			Surgical stages for restorative procedure 2; 3 [*n*(%)]		Indications [*n*(%)]		Robotic platform
Reference (First author, Year)	Total patients	Patients included	Robotic	Laparoscopic	Robotic	Laparoscopic	*p*	Robotic	Laparoscopic	Robotic	Laparoscopic	
Anvari et al. 2004 (25)	20	2	1	1	1 R-STC	1 L-STC	–	/	/	NR	NR	Zeus
D’Annibale et al. 200 (42)	106	3	2	1	2 R-TC	1 L-TC	–	/	/	1 (100) CRC	NR	Da Vinci
Spinoglio et al. 2008 (26)	211	4	1	3	1 R-TC	3 L-TC	–	/	/	NR	NR	Da Vinci
Zimmern et al. 2010 (1)	131	7	7	/	7 R-TC	/	–	/	/	NR	/	NR
Pedraza et al. 2011 (38)	5	5	5	/	5 R-TPC + IPAA	/	–	5 (100): 2	/	3 (60) UC 2 (40) UC + CRC/dysplasia	/	Da Vinci
Byrn et al. 2012 (27)	14	2	1	1	1 Single-Incision R-TPC + EI	1 Single-Incision L-TPC + EI	–	1 (100): 3	1 (100): 3	NR	NR	NR
Domajnko et al. 2012 (39)	27	27	27	/	24 R-TPC + IPAA 3 R-CP + IPAA	/	–	24 (88.8): 2 3 (11.2): 3	/	26 (96.3) UC 1 (3.7) CRC/dysplasia	/	Da Vinci
McLemore et al. 2012 (40)	3	3	3	/	3 R-CP + IPAA	/	–	3 (100): 3	/	3 (100) Toxic UC	/	Da Vinci S/Si
Miller et al. 2012 (28)	34	34	17	17	10 R-CP + IPAA 7 R-CP + EI	10 L-CP + IPAA 7 L-CP + EI	–	17 (100): 3	17 (100): 3	15 (88.2) UC/IC 2 (11.8) CD	14 (82.3) UC/IC 3 (17.7) CD	Da Vinci S
Helvind et al. 2013 (29)	263	3	3	/	3 R-STC	/	–	/	/	3 (100) CRC	/	Da Vinci S/Si
Morelli et al. 2015 (41)	6	6	6	/	6 R-TPC + IPAA	/	–	6 (100): 2	/	1 (16.6) UC 5 (83.4) FAP	/	Da Vinci
Roviello et al. 2015 (42)	4	4	4	/	4 R-TPC + EI	/	–	4 (100): 3	/	4 (100) UC	/	Da Vinci Si
Mark-Christensen et al. 2016 (30)	251	81	81	/	79 R-CP + IPAA 2 R-TPC + IPAA	/	0.14	2 (2.5): 2 79 (97.5): 3	/	81 (100) UC	/	Da Vinci
Moghadamyeghaneh et al. 2016 (31)	26,721	9940	326	9614	326 R-TC	9,614 L-TC	–	/	/	116 (35.6) UC 86 (26.3) CRC 35 (10.8) CD 44 (13.6) BT 19 (5.9) FD 28 (8) Other	3,005 (31.2) UC 1,835 (19) CRC 735 (7.6) CD 369 (3.8) DD 1,160 (12) BT 1,415 (14.7) FD 1,094 (11.3) Other	NR
Rencuzogullari et al. 2016 (32)	42	42	21	21	4 R-TPC +/− IPAA 17 R-CP +/− IPAA	4 l-TPC +/− IPAA 17 l-CP +/− IPAA	>0.99	4 (19): 2 17 (81):3	4 (19): 2 17 (81):3	17 (81) UC 4 (19) CD	17 (81) UC 4 (19) CD	NR
Jimenez-Rodriguez et al. 2018 (7)	23	23	15	8	11 R-TC + IRA 1 R-TPC + IPAA 1 R-TC + EI 2 R-TPC + EI	8 L-TC + IRA	0.91	NR	/	5 (33) FAP 5 (33) LS 3 (20) CRC 2 (13) UC	4 (50) FAP 2 (25) LS 2 (25) CRC	Da Vinci Xi
Marino et al. 2018 (33)	32	32	16	16	16 R-TPC + IPAA	16 L-TPC + IPAA	–	NR	NR	16 (100) IBD	16 (100) IBD	NR
Elias et al. 2019 (34)	105	105	33	72	26 R-TPC + IPAA 7 R-CP + IPAA	51 L-TPC + IPAA 21 L-CP + IPAA	–	26 (78.7): 2 7 (21.2): 3	51 (70.8): 2 21 (29.1): 3	27 (79) UC 1 (3) UC + CRC 5 (15) FAP	65 (90) UC 2 (3) UC + CRC 5 (7) FAP	NR
Hamzaoglu et al. 2019 (43)	10	10	10	/	10 R-TPC + IPAA	/	–	10 (100): 2	/	8 (80) UC 2 (20) CRC/dysplasia	/	Da Vinci Xi
Lightner et al. 2019 (39)	132	132	74	58	37 R-TPC + IPAA 37 R-CP + IPAA	56 L-TPC + IPAA 2 L-CP + IPAA	**<0.001**	37 (50): 2 37 (50): 3	56 (96.5): 2 2 (3.5): 3	59 (79.9) UC/IC 10 (13.5) CRC/dysplasia 5 (6.8) FAP	28 (48.8) UC/IC 14 (24.1) CRC/dysplasia 16 (27.6) FAP	Da Vinci
Ozben et al. 2019 (36)	82	82	26	56	15 R-TC 11 R-STC	31 L-TC 25 L-STC	0.84	/	/	12 (46.2) CRC 14 (53.9) BT	20 (35.7) CRC 36 (64.3) BT	Da Vinci Xi
Hollandsworth et al. 2020 (44)	37	37	37	/	21 R-STC 16 R-TPC +/− IPAA	/	–	16 (100): 2	/	22 (59.5) UC 5 (13.5) CD 2 (5.4) CRC 1 (2.7) FD 6 (16.2) FAP	/	Da Vinci Xi
Kim et al. 2021 (37)	56	56	20	36	8 R-TC 12 R-TPC	8 L-TC 28 L-TPC	0.219	NR	NR	12 (60) CRC 6 (30) FAP 3 (15) UC	18 (50) UC 14 (39) FAP 2 (6) CRC 2 (6) FD	Da Vinci Si/Xi
Total (sum or weighted mean)	28,315	10,640	736	9,904	36 R-STC 371 R-TC 166 R-TPC 163 R-CP	26 L-STC 9,665 L -TC 156 L -TPC 57 L -CP	–	122 (40.6): 2 178 (59.3):3	11 (16): 2 58 (84): 3	384 (53.1) UC 135 (18.6) CRC 58 (8) BT 46 (6.3) CD 27 (3.7) FAP 20 (2.7) FD 16 (2.2) IBD 5 (0.7) LS 3 (0.4) Toxic UC 28 (3.8) Other	3,147 (31.8) UC 1,875 (18.9) CRC 1,417 (14.3) FD 745 (7.5) CD 1,196 (12.1) BT 369 (3.7) DD 29 (0.2) FAP 2 (0.02) LS 16 (0.1) IBD 1,094 (11) Other	–

R, robotic; L, Laparoscopic; STC, subtotal colectomy; TC, total colectomy; CP, completion proctectomy; TPC, total proctocolectomy; IPAA, ileal pouch-anal anastomosis; EI, terminal ileostomy; LI, loop ileostomy, IRA, ileo-rectal anastomosis; UC, ulcerative colitis; IC, indeterminate colitis; CRC, colorectal cancer; CD, Crohn's disease; FAP, familial adenomatous polyposis; BT, benign tumor; FD, functional disorder; LS, Lynch syndrome; IBD, intestinal bowel disease; DD, diverticular disease. Significant *p* values are indicated in bold.

#### 3.2.3. Robotic surgical approach

##### 3.2.3.1. Subtotal colectomy

Four studies reported the outcomes of 36 patients who underwent STC (28, 32, 39, 47) (Table 4). The first literature report of a robotic-assisted STC was published by Anvari et al. in 2004 (28), performed by using the Zeus Microwrist System (Computer Motion, Santa Barbara, CA) with a robotic/laparoscopic hybrid approach. Most other cases (32 interventions; 88.8%) were performed with a totally robotic technique and operated with the Da Vinci Xi robotic platform with a single docking approach and two boom placements.

**Table 4:** Robotic multiquadrant surgery type.

Type of surgery (No. of Patients)	Surgical strategy	No. of Patients [*n* (%)]	Robotic platform (No. of procedures)	No. of Docking (Boom placements)	Reference
Subtotal colectomy (36)	Hybrid procedure	1 (2.7%)			Anvari et al. (25) Helvind et al. (29) Ozben et al. (36) Hollandsworth et al. (44)
	Colon and mesentery mobilisation: Robotic Mesenteric division: Laparoscopic	*1*	Zeus (1)	1	
	Totally robotic	32 (88.8%)	Da Vinci Xi (32)	1 (2)	
	NR	3 (8.3%)	Da Vinci S/Si (3)	NR	
Total colectomy (371)	Totally robotic	37 (9.9%)	Da Vinci (2) Da Vinci Xi (35)	2 1 (2)	D’Annibale et al. (2) Spinoglio et al. (26) Zimmern et al. (1) Moghadamyeghaneh et al. (31) Jimenez-Rodriguez et al. (7) Ozben et al. (36) Kim et al. (37)
	NR	334 (90.1%)	NR (334)	NR	
Total proctocolectomy (166)	Hybrid procedure	78 (46.9%)			Pedraza et al. (38) Byrn et al. (27) Domajnko et al. (39) Morelli et al. (41) Roviello et al. (42) Mark-Christensen et al. (30) Rencuzogullari et al. (32) Jimenez-Rodriguez et al. (7) Marino et al. (33) Elias et al. (34) Hamzaoglu et al. (43) Lightner et al. (35) Hollandsworth et al. (44) Kim et al. (37)
	Colectomy: Laparoscopic Proctectomy: Robotic	*35*	Da Vinci (31) NR (4)	1 1	
	Colectomy: Hand-assisted laparoscopy Proctectomy: Robotic	*6*	Da Vinci (6)	1	
	Colectomy: NR Proctectomy: Robotic	*37*	Da Vinci (37)	1	
	Totally robotic	45 (27.1%)	Da Vinci Si (5) Da Vinci Xi (30) Da Vinci Xi (10)	1 1 (2) 1 (3)	
	NR	42 (25.3%)	NR (42)	NR	
Completion Proctectomy (163)	Hybrid procedure	156 (95.7%)			Domajnko et al. (39) McLemore et al. (40) Miller et al. (28) Mark-Christensen et al. (30) Rencuzogullari et al. (32) Elias et al. (34) Lightner et al. (35)
	Colectomy: Laparoscopic (previous intervention) Proctectomy: Robotic	*119*	Da Vinci (82) Da Vinci S/Si (3) Da Vinci S (17) NR (17)	1 1 1 1	
	Colectomy: NR Proctectomy: Robotic	*37*	Da Vinci (37)	NR	
	NR	7 (4.3%)	NR	NR	

##### 3.2.3.2. Total colectomy

Seven studies analyzed 371 robotic TC procedures (1, 2, 7, 29, 34, 39, 40) (Table 4). Most interventions (334; 92%) were described in a retrospective study based on a national database with little information on the technique and the surgical platform. The remaining 37 procedures were performed using a fully robotic approach: 2 with a non-specified da Vinci platform with 2 dockings and 37 with the da Vinci Xi platform with a single docking and two boom placements.

##### 3.2.3.3. Total proctocolectomy

Overall, 166 TPC were described in 14 studies (7, 30, 33, 35–38, 40–42, 44–47) (Table 4). A hybrid approach was defined as a laparoscopically performed colectomy and a robotic proctectomy for half the cases (78; 46.9%). When reported, the da Vinci robotic platform was used for the hybrid technique. In 45 (27.1%) patients, a totally robotic technique for TPC was described, 40 procedures were performed using the da Vinci Xi platform with a single docking and two or three boom placements in 30 and 10 patients, respectively. In two series, 5 patients underwent a totally robotic TPC with a da Vinci Si platform with a single docking approach (40, 45). The technique used to perform the colectomy in hybrid procedure TPC was laparoscopy in 35 patients and hand-assisted laparoscopy in 6 patients; the technique was unreported in the remaining 37 procedures.

##### 3.2.3.4. Completion proctectomy

In all described cases (156 patients) reported in 7 studies (31, 33, 35, 37, 38, 42, 43) (Table 4), the robotic CP was part of a hybrid approach preceded by a colectomy performed laparoscopically (119 patients) or an undescribed technique (37 patients). In none of the reported cases, the colectomy was performed with a robotic approach. Da Vinci S and Si robotic platforms were used in the reported cases.

Only two studies included CP exclusively (20 patients) (31, 43). All other cases were a part of a series of TPC and CP.

#### 3.2.4. Total and subtotal colectomy

##### 3.2.4.1. Operative outcomes

Six cohort studies (2, 7, 28, 29, 34, 39, 40) and 3 case series were considered (1, 32, 47) in the subgroup of the TC and STC. All cohort studies compared robotic and laparoscopic procedures. Only one study compared the robotic surgical outcome with laparoscopic and open procedures (34). In total, 407 robotic procedures (36 STC and 371 TC) were compared with 9,691 laparoscopic (26 STC and 9,665 TC) procedures.

**Figure 2:**
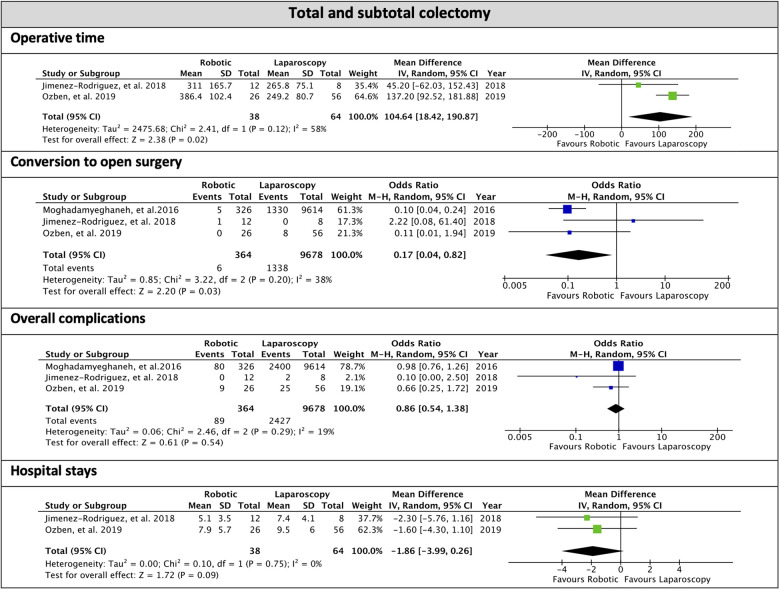
Subgroup of total and subtotal colectomy, forest plots of the overall analysis.

Weighted mean operative time was 331.5 min for the robotic approach and 251.1 for the laparoscopic approach. The MD was 104.64 min in favor of laparoscopy (95% CI, 18.42, 190.87; *p* = 0.02; *I*^2 ^=^ ^58%). Conversion to open surgery was reported in 1.7% of patients who underwent robotic surgery and 13.8% in the laparoscopic procedure. The overall OR for conversion in TC and STC was 0.17 (95% CI, 0.04–0.82; *p* = 0.03) in favor of robotic with moderate heterogeneity (*I*^2 ^=^ ^38%). Anastomosis was fashioned in 37 out of 40 patients in the robotic group and all 38 patients in the laparoscopic group. In the robotic procedures, all anastomoses were stapled, while in laparoscopic procedures, 12.8% were hand-sewn. The intraoperative complication rate was 6.25% in the laparoscopic approach, whereas no intraoperative complication occurred for the robotic approach (Tables 5, Figure 2).

**Table 5:** Total and subtotal colectomy—operative outcomes.

First author, Year	Number and type of operation		Mean age [year(SD)]		Male [*n* (%)]		Mean BMI [kg/m^2^ (SD)]			Operative time [min (SD)]			Blood loss [ml) (SD)]			Conversion to open [*n* (%)]			Anastomosis [*n* (%)]			Ileostomy [*n* (%)]			Intraoperative complication [*n* (%)]		
	R	L	R	L	R	L	R	L	*p*	R	L	*p*	R	L	*p*	R	L	*p*	R	L	*p*	R	L	*p*	R	L	*p*
Anvari et al. 2004 (25)	1 R-STC	1 L-STC	NR	NR	NR	NR	NR	NR	–	NR	NR	–	NR	NR	–	0	0	–	NR	NR	–	NR	NR	–	0	NR	–
D’Annibale et al. 2004 (2)	2 R-TC	1 L-TC	NR	NR	NR	NR	NR	NR	–	NR	NR	–	NR	NR	–	0	NR	–	NR	NR	–	NR	NR	–	0	NR	–
Spinoglio et al. 2008 (26)	1 R-TC	3 L-TC	NR	NR	NR	NR	NR	NR	–	NR	NR	–	NR	NR	–	0	NR	–	NR	NR	–	NR	NR	–	0	NR	–
Zimmern et al. 2010 (1)	7 R-TC	/	41.6	/	2 (28)	/	25.6	/	–	454.2	/	–	228.6	/	–	1 (14.2)	/	–	NR	/	–	NR	/	–	0	/	–
Helvind et al. 2013 (29)	3 R-STC	/	NR	/	NR	/	NR	/	–	NR	/	–	NR	/	–	NR	/	–	NR	/	–	1 EC	/	–	NR	/	–
Moghadamyeghaneh et al. 2016 (31)	326 R-TC	9,614 L-TC	48 (18)	48 (17)	177 (51.1)	4,427 (46.1)	NR	NR	–	NR	NR	–	NR	NR	–	5 (1.5)	1,330 (13.3)	**<0.01**	NR	NR	–	NR	NR	–	NR	NR	–
Jimenez-Rodriguez et al. 2018 (7)	12 R-TC	8 L-TC	52^a^ (20–69^b^)	55^a^ (24–65^b^)	6 (40)	4 (50)	26^a^ (21–40^b^)	25^a^ (21–38^b^)	0.65	243^a^ (169–556^b^)	263^a^ (180–352^b^)	0.97	50^a^ (5–300^b^)	100^a^ (10–300^b^)	0.08	1 (8.3)	0	0.99	11 (81.7) St	7 (87) St 1 (13) Hs	0.99	1 EI	0	0.26	0	0	–
Ozben et al. 2019 (36)	15 R-TC 11 R-STC	31 L-TC 25 L-STC	51.3 (15.4)	56.2 (18.1)	18 (69.2)	36 (64.3)	24.6 (4.5)	25.1 (4.7)	0.72	386.4 (102.4)	249.2 (80.7)	**<0.001**	165.7 (119.1)	197 (120.9)	0.44	0	8 (14.3)	0.051	26 (100) St	41 (73.2) St 7 (12.5) Hs	0.11	2 (7.7) LI	4 (7.1) LI	0.63	0	4 (7.1)	0.30
Hollandsworth et al. 2020 (44)	21 R-STC	/	40.1 (18–79^b^)	/	17 (45.9)	/	24 (13.6–39.3^b^)	/	–	276.8 (119.4)	/	–	NR	/	–	0	/	–	NR	/	–	NR	/	–	0	/	–
Kim et al. 2021 (37)	8 R-TC	8 L-TC	48 (20)	44 (18)	NR	NR	23.7	22.7		NR	NR	–	NR	NR	–	NR	NR	–	NR	NR	–	NR	NR	–	NR	NR	–
Total (sum or weighted mean)	36 R-STC 371 R-TC Total 407	26 L-STC 9,665 L-TC Total 9,691	47.7	48	218 (60)	4,467 (46.1)	22.1	25.4	–	331.5	251.1	–	149.8	188	–	7 (1.76)	1,338 (13.8)	–	37 (100) St	48 (87.2) St 7 (12.8) Hs	–	1 (25) EC 1 (25) EI 2 (50) LI	4 (100) LI	–	0	4 (6.25)	–

R, robotic; L, Laparoscopic; STC, subtotal colectomy; TC, total colectomy; CP, completion proctectomy; TPC, total proctocolectomy; IPAA, ileal pouch-anal anastomosis; EI, terminal ileostomy; LI, loop ileostomy, IRA, ileo-rectal anastomosis; St, stapled; Hs, handsewn.

^a^ Median.

^b^ Range.

Significant *p* values are indicated in bold.

##### 3.2.4.2. Post-operative outcomes

Postoperative complications occurred in 28.4% of the robotic and 25.7% of laparoscopic procedures. The overall OR for postoperative complications was 0.86 (95% CI, 0.54, 1.38; *p*^ ^=^ ^0.54) with low heterogeneity (*I*^2 ^=^ ^19%).

Anastomotic leakage was reported only in 2 cohort studies (7, 39) and one case series (47); it occurred in 1.6% of robotic and 6.2% of laparoscopic procedures. Intrabdominal abscess followed 2.0% of robotic, 1.89% of laparoscopic interventions. Reoperation was necessary in 4.2% of robotic and 10.9% of laparoscopic procedures.

Hospital stay was reported in 4 studies on the robotic (1, 7, 39, 47) and only 2 studies on the laparoscopic approach (7, 39). The meta-analysis showed an overall MD of 1.86 days between robotic and laparoscopic surgery (95% CI, −3.99, 0.26; *p* = 0.09) with moderate heterogeneity (*I*^2 ^=^ ^38%).

Overall, the readmission rate was 30.3% and 10.9% in robotic and laparoscopic surgery, respectively.

No postoperative mortality was reported after robotic procedures, whereas an overall postoperative mortality rate of 0.85% was described for laparoscopic surgery (Table 6, Figure 2).

**Table 6:** Total and subtotal colectomy—post-operative outcomes.

First author, Year	Number and type of operation		Overall complications [*n* (%)]			Clavien-Dindo grade [*n* (%)]		Anastomotic leak [*n* (%)]			Abscess [*n* (%)]			SSI [*n* (%)]			Reoperation [*n* (%)]			Hospital stays [days (SD)]			Readmission [*n* (%)]			Post-operative mortality [*n* (%)]		
	R	L	R	L	*p*	R	L	R	L	*p*	R	L	*p*	R	L	*p*	R	L	*p*	R	L	*p*	R	L	*p*	R	L	*p*
Anvari et al. 2004 (25)	1 R-STC	1 L-STC	NR	NR	–	NR	NR	NR	NR	–	NR	NR	–	NR	NR	–	0	NR	–	NR	NR	–	NR	NR	–	NR	NR	–
D’Annibale et al. 2004 (2)	2 R-TC	1 L-TC	NR	NR	–	NR	NR	NR	NR	–	NR	NR	–	NR	NR	–	0	NR	–	NR	NR	–	NR	NR	–	NR	NR	–
Spinoglio et al. 2008 (26)	1 R-TC	3 L-TC	NR	NR	–	NR	NR	NR	NR	–	NR	NR	–	NR	NR	–	0	NR	–	NR	NR	–	NR	NR	–	NR	NR	–
Zimmern et al. 2010 (1)	7 R-TC	/	4 (57.1)	/	–	NR	/	NR	/	–	NR	/	–	1 (14.2)	/	–	0	/	–	5.3	/	–	5 (71.4)	/	–	0	/	–
Helvind et al. 2013 (29)	3 R-STC	/	NR	/	–	NR	/	NR	/	–	NR	/	–	NR	/	–	NR	/	–	NR	/	–	NR	/	–	NR	/	–
Moghadamyeghaneh et al. 2016 (31)	326 R-TC	9,614 L-TC	80 (23.9)	2,400 (24)	0.99	NR	NR	NR	NR	–	5 (1.5)	180 (1.8)	0.72	14 (4.3)	360 (3.6)	0.53	NR	NR	–	NR	NR	–	NR	NR	–	0	80 (0.8)	0.10
Jimenez-Rodriguez et al. 2018 (7)	12 R-TC	8 L-TC + IRA	0	2 (25)	0.11	–	NR	0	0	–	0	0	–	0	0	–	0	0	–	4^a^ (2–10^b^)	6^a^ (4–12^b^)	**0.04**	3 (20)	0	0.18	0	0	–
Ozben et al. 2019 (36)	15 R-TC 11 R-STC	31 L-TC 25 L-STC	9 (34.6)	25 (44.6)	0.90	1: 4 (15.4) 2: 2 (7.7) 3: 2 (7.7) 4: 1 (3.8)	1: 10 (17.9) 2: 8 (14.3) 3: 5 (8.9) 4: 2 (3.6)	1 (3.8)	4 (8.3)	>0.99	1 (3.8)	3 (5.4)	>0.99	4 (15.4)	5 (8.9)	0.45	2 (7.7)	7 (12.5)	0.71	7.9 (5.7)	9.5 (6.0)	0.08	5 (19.2)	7 (12.5)	0.51	0	0	–
Hollandsworth et al. 2020 (44)	21 R-STC	/	5 (13.5)	/	–	NR	/	0	/	–	3 (8.1)	/	–	2 (5.4)	/	–	1 (2.7)	/	–	5 (4–6)	/	–	7 (18.9)	/	–	0	/	–
Kim et al. 2021 (37)	8 R-TC	8 L-TPC	NR	NR	–	NR	NR	NR	NR	–	NR	NR	–	NR	NR	–	NR	NR	–	NR	NR	–	NR	NR	–	0	0	–
Total (sum or weighted mean)	36 R-STC 371 R-TC Total 407	26 L-STC 9665 L-TC Total 9,691	98 (28.4)	2,427 (25.7)	–	–	–	1 (1.6)	4 (6.2)	–	8 (2.0)	183 (1.89)	–	21 (5.3)	365 (3.7)		3 (4.2)	7 (10.9)		6.19	9.2	–	20 (30.3)	7 (10.9)		0	80 (0.8)	–

R, robotic; L, Laparoscopic; STC, subtotal colectomy; TC, total colectomy; CP, completion proctectomy; TPC, total proctocolectomy; IPAA, ileal pouch-anal anastomosis; EI, terminal ileostomy; LI, loop ileostomy, IRA, ileo-rectal anastomosis; St, stapled; Hs, handsewn.

Significant *p* values are indicated in bold.

^a^ Median.

^b^ Range.

#### 3.2.5. Total proctocolectomy and completion proctectomy

##### 3.2.5.1. Operative outcomes

In the subgroup of TPC and CP, 8 cohort studies (30, 31, 33, 35–38, 40) and 8 case series were considered (7, 41–47). One cohort study used a series of open procedures (33), whereas all the others used laparoscopic series to compare the outcomes of robotic surgery.

In total, 329 robotic procedures (166 TPC and 163 CP) were compared with 213 laparoscopic (156 TPC and 57 CP) procedures.

In 59.3% of the robotic and 63.3% of the laparoscopic procedures, a 3-stage strategy was adopted (Table 3).

No differences were found in the surgical strategy; 59.3% of patients in the robotic group had a three-stage strategy versus 63.4% in the laparoscopic group (Table 3). The overall OR to perform a three-stage procedure was 2.55 (95% CI, 0.24, 27.60; *p* = 0.44; *I*^2 ^= 90%) with no differences between robotic and laparoscopy. In the robotic surgery group, restorative IPAA was performed in 94.8% of patients; in the remaining patients, an ileorectal anastomosis and an end-ileostomy were performed in 0.9% and 3.7%, respectively. In the laparoscopic group, an IPAA was fashioned in 94.8% of patients and an end-ileostomy in 5.5%. Operative time was prolonged in robotic compared to laparoscopic interventions (MD 38.8; 95% CI, 18.7, 59.06; *p* = 0.0002; *I*^2^ = 36%). Blood loss did not significantly differ between the two surgical methods (MD 57.99; 95% CI, −65.2, 181.17; *p* = 0.36; *I*^2^ = 81%). The anastomosis was stapled in 95.7% of the robotic and 98.8% of the laparoscopic group. A loop ileostomy was performed in 92.7% of robotic and 88.6% of laparoscopic patients.

Conversion to open surgery during TPC and CP occurred in 3.4% of the robotic and 4.4% of the laparoscopic group. The overall OR for conversion was 0.45 (95% CI, 0.09, 2.26; *p* = 0.33; *I*^2^ = 0%)showing no differences between the approaches.

**Figure 3:**
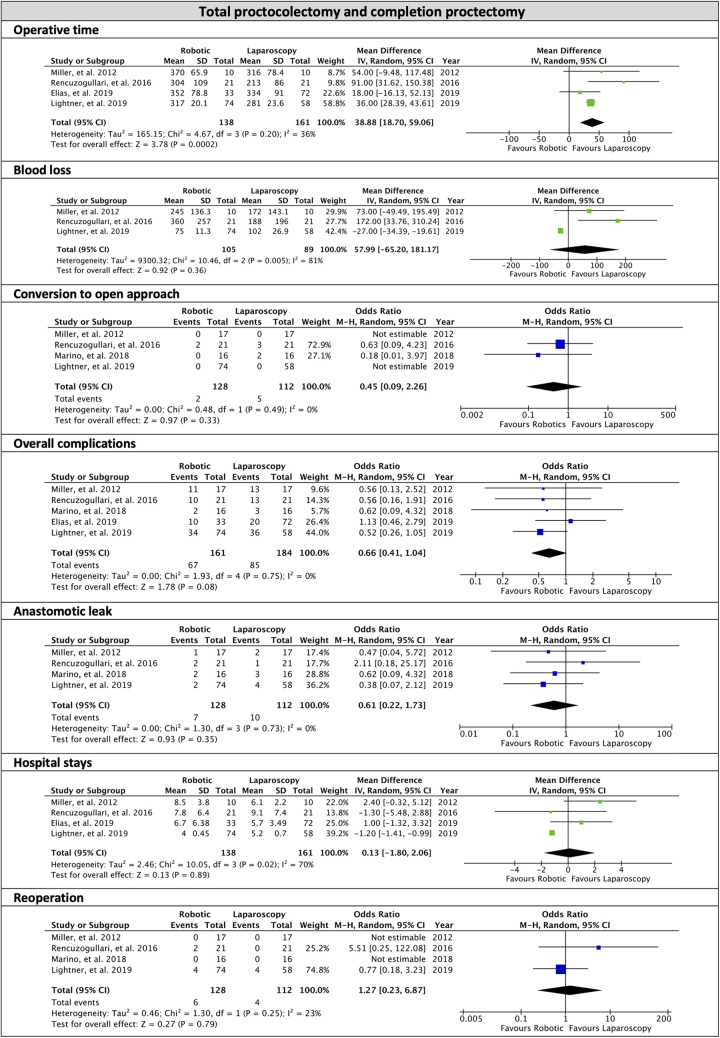
Subgroup of total proctocolectomy and completion proctectomy, forest plots of the overall analysis.

Intraoperative complications occurred in 2% and 2.6% of the robotic and laparoscopic groups, respectively (Table 7, Figure 3).

**Table 7:** Total proctocolectomy and completion proctectomy—operative outcomes.

First author, Year	Type of operation		Mean age [year (SD)]		Male [*n* (%)]		Mean BMI [kg/m^2^ (SD)]			Mean operative time [min (SD)]			Blood loss [ml (SD)]			Conversion to open [*n* (%)]			Restorative technique [*n* (%)]			Anastomosis technique [*n* (%)]			Ileostomy [*n* (%)]	
	R	L	R	L	R	L	R	L	*p*	R	L	*P*	R	L	*p*	R	L	*p*	R	L	*p*	R	L	*p*	R	L
Pedraza et al. 2011 (38)	5 R-TPC	/	45.8 (11.3)	/	2 (40)	/	24.2 (1.9)	/	–	330 (47.4)	/	–	200 (122.5)	/	–	0	/	–	IPAA: 5 (100)	/	–	St: 5 (100)	/	–	LI: 5 (100)	/
Byrn et al. 2012 (27)	1 Single-Incision R-TPC	1 Single-Incision L-TPC	NR	NR	NR	NR	NR	NR	–	NR	NR	–	NR	NR	–	0	NR	–	No: 1 (100)	No: 1 (100)	–	/	/	–	EI: 1 (100)	EI: 1 (100)
Domajnko et al. 2012 (39)	24 R-TPC 3 R-CP	/	46 (16–68)^a^	/	16 (66.6)	/	25 (18.5–30.4)^a^	/	–	407	/	–	154	/	–	NR	/	–	IPAA: 27 (100)	/	–	NR	/	–	NR	/
McLemore et al. 2012 (40)	3 R-CP	/	35,3 (8.1)	/	2 (75)	/	25.6 (2.4)	/	–	436 (87)	/	–	NR	/	–	0	/	–	IPAA: 3 (100)	/	–	St: 3 (100)	/	–	LI: 3 (100)	/
Miller et al. 2012 (28)	17 R-CP	17 L-CP	42.8 (14.8)	43.5 (14.9)	11 (64.7)	9 (52.9)	24.7 (4.3)	25 (2.7)	0.8	CP: 351 (76.3)	CP: 238 (66.4)	**0.03**	CP: 486 (295.4)	CP: 214 (244.5)	0.18	0	0	–	IPAA: 10 (58) No: 7 (42)	IPAA: 10 (58) No: 7 (42)	–	St: 10 (100)	St: 10 (100)	–	LI: 10 (58) EI: 7 (42)	LI: 10 (58) EI: 7 (42)
										CP + IPAA: 370 (65.9)	CP + IPAA: 316 (78.4)	0.14	CP + IPAA: 245 (136.3)	CP + IPAA: 172 (143.1)	0.15											
Morelli et al. 2015 (41)	6 R-TPC	/	26.5 (8)	/	3 (50)	/	24.9 (2.9)	/	–	215 (20.5)	/	–	50.3 (16.4)	/	–	0	/	–	IPAA: 6 (100)	/	–	Hs: 6 (100)	/	–	LI: 6 (100)	/
Roviello et al. 2015 (42)	4 R-TPC	/	30 (24–35)^a^	/	2 (50)		22 (18–26)^a^	/	–	235 (215–255)^a^	/	–	100 (50–200)^a^	/	–	0	/	–	No: 4 (100)	/	–	/	/	–	EI: 4 (100)	/
Mark-Christensen et al. 2016 (30)	7)9 R-CP 2 R-TPC	/	35.4 (13.6)	/	42 (52)	/	23.5 (4)	/	–	284 (67)	/	–	NR	/	–	9 (11.1)	/	–	IPAA: 81 (100)	/	–	St: 79 (97.5) Hs: 2 (2.5)	/	–	LI: 81 (100)	/
Rencuzogullari et al. 2016 (32)	4 R-TPC 17 R-CP	4 L-TPC 17 L-CP	43 (15)	44 (13)	15 (71)	14 (67)	28 (5)	25 (4)	0.09	304 (109)	213 (86)	**0.008**	360 (257)	188 (196)	**0.002**	2 (9.5)	3 (14.2)	>0.99	IPAA: 18 (85.7) No: 3 (14.3)	IPAA: 18 (85.7) No: 3 (14.3)	>0.99	St: 17 (94.4) Hs: 1 (5.6)	St: 17 (94.4) Hs: 1 (5.6)	>0.99	LI: 18 (85.7) EI: 3 (14.3)	LI: 18 (85.7) EI: 3 (14.3)
Jimenez-Rodriguez et al. 2018 (7)	3 R-TPC	/	52 (20–69)^a^	/	6 (40)	/	26 (21–40)^a^	/	–	243 (169–556)^a^	/	–	50 (5–300)	/	–	NR	/	–	IPAA: 1 (33.3) No: 2 (66.6)	/	–	NR	/	–	LI: 1 (33.3) EI: 2 (66.6)	/
Marino et al. 2018 (33)	16 R-TPC	16 L-TPC	NR	NR	NR	NR	NR	NR	–	298	264	**<0.05**	179	288	**<0.05**	0	2 (12.5)	0.15	IPAA: 16 (100)	IPAA: 16 (100)	–	NR	NR	–	NR	NR
Elias et al. 2019 (34)	26 R-TPC 7 R-CP	51 L-TPC 21 L-CP	37.8	37.8	67 (58)	67 (58)	24.5 (16.1–40)^a^	24.5 (16.1–40)^a^	–	333^b^ (235–524)^a^	313^b^ (188–584)^a^	**0.009**	NR	NR	–	NR	NR	–	IPAA: 44 (100)	IPAA: 72 (100)	–	NR	NR	–	NR	NR
Hamzaoglu et al. 2019 (43)	10 R-TPC	/	27^b^ (14–48)^a^	/	5 (50)	/	21^b^ (15–25)^a^	/	–	380^b^ (300–480)^a^	/	–	65^b^ (5–400)^a^	/	–	0	/	–	IPAA: 10 (100)	/	–	St: 10 (100)	/	–	LI: 10 (100)	/
Lightner et al. 2019 (35)	37 R-TPC 37 R-CP	56 L-TPC 2 L-CP	40^b^ (30–50)^c^	40^b^ (30–50)^c^	45 (608)	32 (55.2)	24.5 (3.9)	24.8 (5.7)	0.73	315^b^ (276–365)^c^	281^b^ (235–335)^c^	**0.002**	75^b^ (50–100)^c^	100^b^ (50–175)^c^	**0.002**	0	0	–	IPAA: 74 (100)	IPAA: 58 (100)	–	St: 74 (100)	St: 58 (100)	–	LI: 74 (100)	LI: 58 (100)
Hollandsworth et al. 2020 (44)	16 R-TPC	/	40.1 (18–79)^a^	/	17 (45.9)	/	24.04 (13.67–39.33)	/	–	347.8 (34.3)	/	–	NR	/	–	0	/	–	IPAA: 9 (56.2) IRA: 3 (18.7)	/	–	NR	/	–	LI: 9 (56.2)	/
Kim et al. 2021 (37)	12 R-TPC	28 L-TPC	48 (20)^d^	44 (18)^d^	NR	NR	23.7 (3.8)^d^	22.7 (3.9)^d^	0.49	281 (51)^d^	223 (68)^d^	**0.003**	54 (64)^d^	39 (51)^d^	0.34	0^d^	3 (8)^d^	0.54	IPAA: 12 (100)	IPAA: 28 (100)	–	NR	NR	–	LI: 12 (100)	LI: 28 (100)
Total (sum or weighted mean)	166 R-TPC 163 R-CP 329 Total	156 L-TPC 57 L-CP 213 Total	38.8	38.1	233 (77.6)	122 (72.1)	24.3	24.7	–	321.5	287.7	–	148.9	154.2	–	11 (3.4)	5 (4.4)	–	IPAA: 316 (94.8) IRA: 3 (0.9) No: 14 (4.2)	IPAA: 202 (94.8) No: 11 (5.2)	–	St: 204 (95.7) Hs: 9 (4.3)	St: 85 (98.8) Hs: 1 (1.2)	–	LI: 229 (94.6) EI: 13 (5.4)	LI: 114 (91.2) EI: 11 (8.8)

R, robotic; L, Laparoscopic; CP, completion proctectomy; TPC, total proctocolectomy; IPAA, ileal pouch-anal anastomosis; EI, terminal ileostomy; LI, loop ileostomy, IRA, ileo-rectal anastomosis; St, stapled; Hs, handsewn.

^a^ Range.

^b^ Median.

^c^ Inter quartile range.

^d^ Outcomes reported include together TPC and TC and are not considered for total count and meta-analysis.

Significant *p* values are indicated in bold.

##### 3.2.5.2. Post-operative outcomes

Overall postoperative complications occurred in 47.4% of the robotic and in 46.1% of laparoscopic procedures during TPC and CP, with an OR of 0.66 (95% CI, 0.41, 1.04; *p* = 0.08) and no heterogeneity (*I*^2^ = 0%). Clavien-Dindo grading system was reported in 4 series of robotic surgery (33, 37, 43, 45) and only one of laparoscopy surgery (33, 37); in the robotic group, 72.6% of the complications were classified as grade I–II, and 27.4% were grade III–IV. Anastomotic leakage occurred in 4.9% of robotic and 8.9% of laparoscopic procedures, with an OR of 0.61 (95% CI, 0.22, 1.73; *p* = 0.35) and no heterogeneity (*I*^2 ^= 0%). Reoperation was necessary for 6.7% of robotic and 3.5% of laparoscopic interventions (OR: 1.27; 95% CI, 0.23, 6.87; *p* = 0.79; *I*^2^ = 23%). The MD for hospital stay was 0.13 days (95% CI, −1.80, 2.06; *p* = 0.89) with high heterogeneity (*I*^2^ = 70%). No postoperative mortality was reported in both groups. Readmission was reported in 22% of robotic and 17.8% of laparoscopic patients (Table 8, Figure 3).

**Table 8:** Total proctocolectomy and completion proctectomy—operative and post-operative outcomes.

First author, Year	Type of operation		Intraoperative complication [*n* (%)]			Overall complication [*n* (%)]			Clavien-Dindo grade [*n* (%)]		Anastomotic leak [*n* (%)]			Absess [*n* (%)]			SSI [*n* (%)]			Reoperation [*n* (%)]			Readmission [*n* (%)]			Post-operative mortality [*n* (%)]			Hospital stay [days (SD)]		
	R	L	R	L	*p*	R	L	*p*	R	L	R	L	*p*	R	L	*p*	R	L	*p*	R	L	*p*	R	L	*p*	R	L	*p*	R	L	*p*
Pedraza et al. 2011 (38)	5 R-TPC	/	0	/	–	2 (40)	/	–	NR	/	0		–	1 (20)	/	–	0	/	–	0	/	–	1 (20)	/	–	0	/	–	5.6 (2.6)	/	–
Byrn et al. 2012 (27)	1 Single-Incision R-TPC	1 Single-Incision L-TPC	NR	NR	–	NR	NR	–	NR	NR	NR	NR	–	NR	NR	–	0	0	–	NR	NR	–	NR	NR	–	NR	NR	–	NR	NR	–
Domajnko et al. 2012 (39)	24 R-TPC 3 R-CP	/	1	/	–	7 (25.9)	/	–	NR	/	3 (11)	/	–	2 (7.4)	/	–	0	/	–	3 (11)	/	–	0	/	–	0	/	–	5.8 (3–14)^a^	/	–
McLemore et al. 2012 (40)	3 R-CP	/	1 (33,3)	/	–	3 (100)	/	–	1:1 (33.3) 3a: 2 (66.6)	/	0	/	–	1 (33,3)	/	–	1 (33,3)	/	–	0	/	–	1 (33,3)	/	–	0	/	–	7 (4–8)^a^	/	–
Miller et al. 2012 (28)	17 R-CP	17 L-CP	0	0	NR	11 (64.7)	13 (76.4)	NR	NR	NR	1 (5.8)	2 (11.6)	NR	1 (5.8)	2 (11.6)	NR	1 (5.8)	2 (11.6)	NR	0	0	NR	NR	NR		0	0	NR	CP: 6.4 (1)	CP: 4.1 (0.7)	**0.02**
																													CP + IPAA: 8.5 (3.8)	CP + IPAA: 6.1 (2.2)	0.17
Morelli et al. 2015 (41)	6 R-TPC	/	0	/	–	1 (16.6)	/	–	NR	/	0	/	–	0	/	–	0	/	–	0	/	–	0	/	–	0	/	–	13.2 (7.4)	/	–
Roviello et al. 2015 (42)	4 R-TPC	/	0	/	–	3 (75)	/	–	1–2: 2 (50) 4: 1 (25)	/	0	/	–	0	/	–	1 (25)	/	–	1 (25)	/	–	NR	/	–	0	/	–	6	/	–
Mark-Christensen et al. 2016 (30)	79 R-CP 2 R-TPC	/	1 (1.2)	/	–	57 (70)	/	–	1:15 (19) 2: 23 (28) 3: 18 (22)	/	4 (4.9)	/	–	4 (4.9)	/	–	1 (1.2)	/	–	8 (9.8)	/	–	32 (40)	/	–	0	/	–	9.1 (5)	/	–
Rencuzogullari et al. 2016 (32)	4 R-TPC 17 R-CP	4 L-TPC 17 L-CP	1 (4.7)	1 (4.7)	>0.99	10 (47.6)	13 (61.9)	NR	NR	NR	2 (9.5)	1 (4.7)	>0.99	NR	NR	–	3 (14.2)	1 (4.7)	0.61	2 (9.5)	0	0.11	3 (14.2)	3 (14.2)	>0.99	0	0	–	7.85 (6.41)	9.19 (7.47)	0.39
Jimenez-Rodriguez et al. 2018 (7)	3 R-TPC	/	0	/	–	0	/	–		/	0	/	–	0	/	–	0	/	–	0	/	–	NR	/	–	0	/	–	4 (2–10)	/	–
Marino et al. 2018 (33)	16 R-TPC	16 L-TPC	NR	NR	–	2 (12.5)	3 (18.7)	0.63	NR	NR	2 (12.5)	3 (18.7)	NR	NR	NR	–	NR	NR	–	0	0	–	0	0	–	0	0	–	8.7	9.2	**0.02**
Elias et al. 2019 (34)	26 R-TPC 7 R-CP	51 L-TPC 21 L-CP	NR	NR	–	10 (29)	20 (28)	1	1:7 (21) 2: 3 (9)	1:14 (19) 2: 6 (8)	NR	NR	–	NR	NR	–	NR	NR	–	NR	NR	–	NR	NR	–	NR	NR	–	4^b^ (3–22)^a^	4^b^ (3–17)^a^	**0.02**
Hamzaoglu et al. 2019 (43)	10 R-TPC	/	0	/	–	5 (50)	/	–	1: 5 (50)	/	0	/	–	0	/	–	3 (30)	/	–	0	/	–	0	/	–	0	/	–	6 (4–12)^a^	/	–
Lightner et al. 2019 (35)	37 R-TPC 37 R-CP	56 L-TPC 2 L-CP	NR	NR	–	34 (46)	36 (62)	NR	NR	NR	2 (2.7)	4 (6.9)	0.25	5 (6.8)	9 (15.5)	0.10	5 (6.8)	4 (6.9)	0.97	4 (5.4)	4 (6.9)	0.72	13 (17.6)	14 (24.1)	0.35	0	0	–	4^b^ (3–5)^c^	5^b^ (4–7)^c^	0.05
Hollandsworth et al. 2020 (44)	16 R-TPC +/− IPAA	/	0	/	–	5 (13.5)	/	–	NR	/	0	/	–	3 (8.1)	/	–	2 (5.4)	/	–	1 (2.7)	/	–	7 (18.9)	/	–	0	/	–	5^b^ (4–6)^d^	/	–
Kim et al. 2021 (37)	12 R-TPC	28 L-TPC	NR	NR	–	2 (10)^e^	12 (33)^e^	0.06	1/2^e^	1/2^e^	0^e^	0^e^	–	0^e^	0^e^	–	0^e^	1 (3)^e^	1	0^e^	0^e^	–	NR	NR	–	0^e^	0^e^	1	10 (3)^e^	10 (7)^e^	0.8
Total (sum or weighted mean)	154 R-TPC 163 R-CP 317 Total	128 L-TPC 57 L-CP 185 Total	4 (2)	1 (2.6)	–	150 (47.4)	85 (46.1)	–	1: 30 (38.9) 2: 26 (33.7) 3:20 (25.9) 4:1 (1.3)	1:14 (19) 2: 6 (8)	14 (4.9)	10 (8.9)	–	17 (3.6)	11 (14.9)	–	17 (6.3)	7 (7.2)	–	19 (6.7)	4 (3.5)	–	57 (22)	17 (17.8)		0	0	–	7.8	6.7	–

R, robotic; L, Laparoscopic; CP, completion proctectomy; TPC, total proctocolectomy; IPAA, ileal pouch-anal anastomosis; EI, terminal ileostomy; LI, loop ileostomy, IRA, ileo-rectal anastomosis; St, stapled; Hs, handsewn.

^a^ Range.

^b^ Median.

^c^ Inter quartile range;

^d^ 25–75 percentile.

^e^ Outcomes reported include together TPC and TC and are not considered for total count and meta-analysis.

Significant *p* values are indicated in bold.

#### 3.2.6. Study quality assessments

According to the NOS, the quality of the retrospective cohort studies was 8/9 in 2 studies (33, 34), 7/9 in 7 studies (7, 31, 32, 35, 38–40), 6/9 in 4 studies (2, 28, 29, 37), 5/9 in one study (30) and 4/9 in one study (36).

Based on the quality assessment described by Murad (23), 3 series received a score of 6/8 (41, 43, 47) and 5 received a grade of 5/8 (1, 42, 44–46). Overall, the 23 studies were considered at high risk of bias.

Based on the GRADE system, the overall quality of evidence was rated as low. The study quality and risk of bias of the included studies are summarized in the additional file: **Table Supplementary 3**.

## 4. Discussion

Multiquadrant colorectal procedures represent challenging interventions performed with a totally robotic approach. However, our single-center experience and an appraisal of the literature support the safety and feasibility of robotic TPC, associated with satisfactory postoperative outcomes comparable to laparoscopy. Specifically, for STC and TC, the robotic approach appeared to be associated with a lower conversion rate than laparoscopy. We found a trend in favor of robotic surgery for the duration of hospital stay (for TC and STC procedures) and the overall rate of postoperative complications (for TPC and CP procedures). However, the operative time was longer for robotic procedures than laparoscopy.

Interest in robotic-assisted colorectal surgery has risen exponentially (48) in the last few years, with a consequent increase in the number of original articles and reviews published each year (49). There is a paucity of data on the application of robotic surgery for multiquadrant resections. A recent systematic review examined the outcomes of restorative proctocolectomy, limiting the analysis to robotic proctectomy or proctocolectomy with IPAA (50). Based on 6 comparative studies and 3 case series, the authors preferred the robotic platform for similar (or slightly better) outcomes than laparoscopy (50). The present systematic review updates these findings with the most recent evidence and considers TC and STC procedures performed robotically. Notably, staged procedures were often performed using a hybrid approach, implying that colectomy was often performed laparoscopically, whereas CP used a robotic platform. Even if only the proctectomy was performed with the help of the robotic platform, CP were included in this study as a part of staged multiquadrant robotic procedures. Concerning the small bowel mesentery mobilization in CP, only three studies described the technique used and in all of them the mobilization was performed laparoscopically (31, 33, 43). Interestingly, in the mentioned studies all the surgeries were performed with the old generation robotic platform that could not provide access to all four abdominal quadrants with a single cart positioning (7–9) and this could influence the choice in favor to the laparoscopic approach. This finding was also observed for TPC, which could lead to missing evidence on the potential advantages of an approach over the other because longer operative times may be necessary to allow the shift from laparoscopy to robotic surgery during the same procedure.

Despite this heterogeneity, the overall evidence is based on a not negligible sample, accounting for more than 10,600 patients who underwent a robotic or laparoscopic procedure, suggesting that minimally invasive surgery for multiquadrant colorectal resections is widespread and acceptable. This evolution may be also linked to the introduction of the da Vinci Xi surgical system, characterized by the boom-mounted arms and the ability of the boom to rotate and provide access to all four abdominal quadrants with a single cart positioning (7–9). Most totally robotic procedures used the da Vinci Xi system with a single docking approach. The only difference in the surgical technique was the number of boom placement, which varied from 2 to 3 on some occasions when proctectomy was needed. This strategy is in accordance with Protyniak et al. (8), who demonstrated that the Xi platform can be used for multiquadrant surgery without repositioning the patient-side surgical cart. Recently two comparative studies (7, 39) and two case series (46, 47) evaluated the da Vinci Xi surgical system and multiquadrant surgery for TC, STC, and restorative TPC, using a totally robotic approach. All authors agreed that the robotic platform offered a significant advantage over the previous generations. Likewise, we reported our experience with the technical note to perform totally robotic TPC, using the totally robotic approach (**Supplementary material**).

There is consistent literature indicating that the operative time for robotic multiquadrant procedures is longer than laparoscopy. Only one study showed no differences in the operative time between 15 robotic procedures (TC and TPC) and 8 laparoscopic TC (7). In this study, all the patients were operated using the da Vinci Xi system, and the authors justified these findings, attributing a major advantage of the robotic platform over the previous versions. Morelli et al. demonstrated evidence supporting the advantages of the da Vinci Xi over the da Vinci Si for multiquadrant and combined procedures (51, 52).

The present study findings are limited by the nature of the studies included. The available literature consists of retrospective case series or cohort studies. No RCT was found, and its performance would be extremely hazardous. A high degree of heterogeneity in the surgical techniques was noted, also regarding the different indications for surgery. We tried to overcome this bias by performing separated analysis for procedures including or not a proctectomy. Sensitivity analyses were not performed due to the paucity of data on the subgroups of patients and procedures. Similarly, it was not possible to analyze the outcomes of the alternative surgical strategies for TPC, comparing two- to three-stage surgeries, for example. Four comparative studies reported a combination of two- and three-stage procedures (33, 35, 37, 38), and only one cohort by Lightner et al. reported a significant difference between the procedures (38). However, the meta-analysis showed no differences in the surgical strategy adopted. Outcomes were limited to operative and postoperative variables. Patient-centered outcomes, including urological, sexual dysfunction, and quality of life, remain essentially unexplored (53).

Our experience and a systematic review of the literature suggest that robotic multiquadrant colorectal surgery is safe and effective, with low morbidity and mortality rates. The overall level of evidence merging from the present systematic review was judged as low. The functional outcomes associated with robotic TPC, TC, STC, or CP should be the focus for future studies.

## Data Availability

The original contributions presented in the study are included in the article/**Supplementary Material**, further inquiries can be directed to the corresponding author/s.
