# Supplementary material for: Robotic multiquadrant colorectal procedures: A single-center experience and a systematic review of the literature
Source: Front Surg. 2022 Aug 17;9:991704. doi: 10.3389/fsurg.2022.991704 (PMC9428340; doi:10.3389/fsurg.2022.991704)
Supplement: Supplementary file 1 [file Table_1_v1.docx]

**Supplemental Material. Description of the robotic total proctocolectomy (TPC)**

Patient’s installation and port positioning

The patient is placed in a modified lithotomy position with the legs in Allen stirrups. The robotic cart is placed between the patient’s legs on the median axis. This positioning allows the rotation of the boom in all necessary positions as well as an easy access for the surgeon for the introduction of circular stapler without moving the patient-side surgical cart during the surgery. Only the robotic boom is rotated and manually adjusted in two different positions according to the operational steps: 1) right colectomy and transverse colon mobilization; 2) left colectomy and rectal resection with total mesorectal excision (TME) and ileal pouch-anal anastomosis (IPAA). For the first step, the robotic boom is rotated on the right side of the patient, who is placed in a Trendelenburg position tilted to the left (20°). For the second step, the robotic boom is rotated on the left side of the patient, who remains in the Trendelenburg position but tilted to the right (20°). The visual cart (robotic tower with monitor) remains on the right side of the patient, and it is twisted according to the procedural steps (Figures 1A and 1B).

The pneumoperitoneum is created using an 8-mm robotic port placed by an open approach in the upper right side of the umbilicus. After insufflation with 12 mmHg of CO_2_, the remaining ports are placed. In total, 4 robotic (three 8-mm ports and one 12-mm port) and 2 laparoscopic (5-mm) ports are used. The 4 robotic ports are placed on a diagonal line drawn from the right femoral head (lateral border of the inguinal triangle) to where the left mid clavicular line (MCL) crosses over the left subcostal margin. Ports are placed equally distant (every 8 cm). The first assistant laparoscopic port is placed 2 cm left from the left-side MCL at the level of the left subcostal margin (AirSeal®, Conmed Corp, Utica, NY, USA) and is used for the right and transverse colectomy steps with the robotic camera docked in the second robotic arm (Figure 2A). The second laparoscopic port is used for the left colectomy and rectal resection steps and is placed 2 cm right to the right-side MCL at the level of the umbilicus with the robotic camera docked in the third robotic arm (Figure 2B).

During the procedure, fenestrated bipolar forceps, monopolar curved scissors, tip-up fenestrated grasper, and da Vinci vessel sealer (Intuitive Surgical, Sunnyvale, CA, USA) are used.

Operative steps

*Two-stage procedure*

The TPC is performed following a medial-to-lateral approach with proximal vascular ligation of the colic pedicles. The procedure begins on the right side with entry into the retroperitoneum in the avascular portion of the mesentery between the superior mesenteric artery and the ileo-colic vessels. The mesentery of the colon is lifted from the retroperitoneal place, and the C of the duodenum and the head of the pancreas are identified. The ileo-colic and right colic pedicles are dissected close to their origin and sectioned using Hem-o-loc clips and vessel sealer. The dissection proceeds along the root of the superior mesenteric axis, and the middle colic artery and vein are dissected and clipped. The procedure is followed by the lateral mobilization of the right colon and the dissection of the gastro-colic ligament. This step allows a complete mobilization of right and transverse colon including the splenic flexure.

After rotation of the robotic boom, the left colectomy is performed with a medial-to-lateral approach. The inferior mesenteric artery and vein were dissected at their origin and sectioned using Hem-o-loc clips and vessel sealer followed by the lateral mobilization of left colon.

Then, a sphincter-preserving low rectum resection with TME is performed. The rectal transection is achieved with Endo-Wrist 45-mm stapler using the 12-mm robotic port placed in the right iliac fossa. The first robotic arm is used to pull the rectal stump down and out of the pelvis to provide proper tension on the anterior structures, while a suction device through the laparoscopic assistant port is used to facilitate the dissection. The completion and adequateness of the rectal dissection is checked by digital transanal exam just before transecting the rectum by using 2 to 3 fires of the da Vinci EndoWrist 45-mm stapler (Intuitive Surgical, Sunnyvale, CA, USA).

Following exsufflation, an incision is made 3 cm right to the umbilicus; this incision is used for extracting the surgical specimen, creating an extracorporeal J pouch, and exteriorizing a protective loop ileostomy. After the extraction of colon and rectum, the mesentery of terminal ileum is ligated and dissected; the ileum is dissected using GIA 80-mm stapler 5 cm proximally from the ileo-cecal valve. The complete specimen is sent to histological examination (Figure 3A).

The creation of a 20-cm ileal J pouch is done using GIA 80-mm stapler ^8^. The anvil of circular 29-mm stapler is fixed with purse-string suture in the distal angle of the J pouch. Near-infrared wavelength excited indocyanine green (0.1 mg/kg, at 2.5 mg/1 ml, intravenous, 40 s) is injected to assess the blood supply of the intestinal tissue while the robot was put in the Firefly mode. The test confirms the viability of the complete ileal pouch. The ileum is reintroduced into the abdominal cavity before re-insufflation. With a circular 29-mm stapler introduced transanally, an ileo-anal end-to-end anastomosis is constructed. The tissue perfusion and viability are retested by re-activating the Firefly mode (Figure 3B).

After the positioning of a drain in the pelvis and the complete exsufflation, a protective loop ileostomy is exteriorized using the incision close to the umbilicus (Figure 3C).

*Three-stage procedure*

In the first part of the three-stage procedure total colectomy is performed with the same technique. Once the colon is totally mobilized, following exsufflation, an incision is made 3 cm left to the umbilicus; this incision is used for extracting the colon. The mesentery of terminal ileum and the sigmoid colon are ligated and dissected; the ileum is dissected using GIA 80-mm stapler 5 cm proximally from the ileo-cecal valve; the sigmoid colon is dissected proximal to the sacral promontory. A double barreled ileo-sigmoid ostomy is created.

In the second operation the ileo-sigmoid ostomy is dissected, wound protector with laparoscopic cap (Alexis; Applied) is placed and a pneumoperitoneum is created.

The robotic cart is placed on the left side of the patient and the tower cart is placed between the patient’s legs.

The 4 robotic (three 8-mm and one 12-mm) ports and the right side assistant laparoscopic port are placed in the same place of the first operation.

Rectal resection with TME and IPAA are performed using the same technique of the two-stage technique, the same port placement and docking. Once TME is performed and after exsufflation surgical specimen is removed from the incision of the previous ostomy. A IPAA is done with the same technique described.

After the positioning of a drain in the pelvis and the complete exsufflation, a protective loop ileostomy is exteriorized using the incision close to the umbilicus

**Figure 1.**

**(a) Operating room set up during the right colectomy and transverse colon mobilization steps of the total proctocolectomy.** The robotic cart is placed between the patient’s leg with the robotic boom rotated 90° to the left.

**(b) Operating room set up during the left colectomy and rectal resection steps of the total proctocolectomy.** The robotic cart remains between the patient’s leg with the robotic boom rotated 90° to the right.

**Figure 2.**

**(a) Port placement and target anatomy positioning for the right colectomy and transverse colon mobilization** **steps of** **the total proctocolectomy.** Four robotic ports were used: one 12-mm port (port #1) and three 8-mm ports (ports #2,3,4). The robotic camera was in the second port. One laparoscopic port (A) was placed on the left side for the AirSeal® (Conmed Corp, Utica, NY, USA).

**(b) Port placement and target anatomy positioning for the left colectomy and rectal resection steps of the total proctocolectomy.** Four robotic ports were used: one 12-mm port (port #4) and three 8-mm ports (ports #1,2,3). The robotic camera was in the third port. One laparoscopic port (A) was placed on the right side.

**Figure 3.**

**(a) The surgical specimen.**

**(b) Indocyanine green fluorescence just after the IPAA construction.**

**(c) Protective loop ileostomy.**
